# Whole-Genome Sequencing of Russian Neisseria Gonorrhoeae Isolates Related to ST 1407 Genogroup

**Published:** 2018

**Authors:** A. A. Kubanov, A. V. Runina, A. V. Chestkov, A. V. Kudryavtseva, Y. A. Pekov, I. O. Korvigo, D. G. Deryabin

**Affiliations:** State Research Centre of Dermatovenerology and Cosmetology, Korolenko Str., 3/6, Moscow, 107076 , Russia; Engelhardt Institute of Molecular Biology, Russian Academy of Sciences, Vavilova Str., 32, Moscow, 119991, Russia; Ksivalue Data Analysis Studio, Leninsky Ave., 30A, Moscow, 117628, Russia

**Keywords:** Neisseria gonorrhoeae, whole genome sequencing, genetic determinants of antimicrobial drug resistance, phylogenetic analysis

## Abstract

The whole-genome sequencing data of three *N. gonorrhoeae *strains isolated in the Russian Federation in 2015 are presented. According to the NG-MAST protocol, these strains are related to the globally spread ST 1407 genogroup. The analysis of their resistomes showed the absence of *ermA/B/C/F *genes and the presence of wild-type alleles of *rpsE, rrs, rrl, rplD, rplV, macAB, *and *mefA *genes, and these patterns explain the susceptibility of the sequenced strains to aminocyclitols (spectinomycin) and macrolides (azithromycin). Conjugative resistance determinants (*blaTEM, tetM*) were absent in the genomes, and the *penC/ pilQ*, *parE, *and *norM *alleles were shown to be wild-type, whereas single or multiple nucleotide substitutions were identified in the genes encoding targets for β-lactams (*ponA, penA*), tetracyclines (*rpsJ*), and fluoroquinolones (*gyrA, parC*). The additional mutations were found in *porB *gene and the promoter of *mtrR *gene, which nonspecifically reduced the susceptibility to antimicrobials due to the membrane permeability decrease and efflux pump overexpression. The diversity of mutations observed in the analyzed genomes prompted a revision of the phylogenetic relationships between the strains by comparing more than 790 groups of housekeeping genes. A high homology between the *N. gonorrhoeae *ST 1407 and *N. gonorrhoeae *ST 12556 genomes was confirmed; the latter had probably diverged from a common ancestor as a result of single mutation events. On the other hand, *N. gonorrhoeae *ST 12450 was an example of phenotypic convergence which appeared in the emergence of new drug resistance determinants that partially coincide with those of the ST 1407 genogroup.

## INTRODUCTION

Gonorrhoea remains one of the four most widely spread sexually transmitted diseases. In 2012, its incidence among reproductive-age individuals reported by the WHO was 78.3 million new cases [1]. A total of 27,056 cases of gonococcal infection in 2015 in Russia, corresponding to 18.5 cases per 100,000, were reported [2]. Whereas in Russia these figures annually decrease by 10–20%, incidence of the infection worldwide continues to increase steadily.

The increase in gonorrhoea incidence is due to the appearance and spread of epidemic high-risk clones of *Neisseria gonorrhoeae *which possess multiple antibiotic resistance, including resistance to third-generation cephalosporins, the drugs of choice for treatment of the infection [3]. Currently, the genetic variant referred to ST 1407 [5] in accordance with the NG-MAST (*Neisseria gonorrhoeae *Multi-Antigen Sequence Typing) protocol [4] has gained much significance. By 2010, this sequence type had been reported in 20 out of 21 states of the European Union, accounting for over 10% of isolates in 13 EU states (including Austria, Belgium, Italy, the Netherlands, Portugal, Romania, Slovenia, Spain, and the UK) [6]. Genetic analysis revealed multiple antibiotic resistance determinants in *N. gonorrhoeae *ST 1407 [7], and that explains the increasing ineffectiveness of gonococcal infection therapy [3, 5].

The epidemic significance of ST 1407 is additionally determined by the existence of phylogenetically related molecular types of *N. gonorrhoeae *that are 99% amino acid identical to *porB *or *tbpB *genes used for NG-MAST typing and are, therefore, considered to belong to the ST 1407 genogroup. Considering this, the percentage of *N. gonorrhoeae *isolates in the EU states is as high as 23% [6], while one of the representatives of this genogroup, ST 4378, is the predominant sequence type in Taiwan [8].

Cases of gonococcal infection caused by *N. gonorrhoeae *ST 1407 are sporadic in Russia, being reported in cities with intensive touristic (Moscow) or economic (Kaluga and Murmansk) migration from the EU states [9]. Earlier, we reported few potential representatives of the genogroup ST 1407 detected in Russia for the first time [10]. This study aimed to perform an in-depth genome characterization of these isolates using whole-genome sequencing, which has recently become a high-demanded tool for analyzing the molecular mechanisms of antibiotic resistance and phylogeny of pathogenic microorganisms.

## MATERIALS AND METHODS

***N. gonorrhoeae *strains**

Three *N. gonorrhoeae *strains as objects of the study were isolated from patients with a diagnosis of gonococcal infection of the lower genitourinary tract (ICD-10 diagnosis code A54.0) in 2015 and were deposited in a specialized collection of the State Research Center of Dermatovenereology and Cosmetology (Ministry of Health of the Russian Federation) with the identification codes 20/15/004, 41/15/003, and 19/15/005.

The *N. gonorrhoeae *strain 20/15/004 was isolated from a 41-year -old male patient in Kaluga; NG-MAST type referred to the epidemically high-risk genogroup ST 1407 (*porB *allele 908 and *tbpB *allele 110 according to the NG-MAST database nomenclature) [4]. *N. gonorrhoeae *strain 41/15/003 was obtained from a 19-year-old male patient in Tomsk and characterized as a novel sequence type ST 12556 carrying a previously unknown combination of six *tbpB *alleles and *porB *allele 971; the latter exhibited 99.97% homology with ST 1407. *N. gonorrhoeae *strain 19/15/005 was isolated from a 31-year-old female patient in Omsk and was also characterized as being a novel ST 12450 NG-MAST type with the combination of *porB *allele 931 and the previously unknown *tbpB *allele 2097 and with a 99.74% homology and with six other alleles of this gene described earlier. An analysis of these strains within sample consisting of 124 *N. gonorrhoeae *cultures conducted by comparing the fusion sequences of the *porB *and *tbpB *genes using the MEGA 6 (Molecular Evolutionary Genetics Analysis version 6.0) software [11] demonstrated that they belong to one genogroup of ST 1407 [10].

**Table 1:** Parameters of susceptibility of the analyzed N. gonorrhoeae strains to antimicrobials and their interpretation according to the Methodology Guidelines MUK 4.2.1890–04*

Antimicrobial (zones of susceptibility – S, moderate susceptibility – MS, and resistance – R; µg/ml)	MIC, µg/ml		
	20/15/004	41/15/003	19/15/005
Benzylpenicillin (S ≤ 0.06; MS = 0.12–1; R ≥ 2)	0.5 (MS)	2 (R)	1 (MS)
Ceftriaxone (S ≤ 0.25; MS ≥ 0.25)	0.015 (S)	0.03 (S)	0.015 (S)
Tetracycline (S ≤ 0.25; MS = 0.5–1; R ≥ 2)	2 (R)	4 (R)	2 (R)
Spectinomycin (S ≤ 32; MS = 64; R ≥ 128)	32 (S)	32 (S)	16 (S)
Azithromycin** (S ≤ 0.25; MS = 0.5; R ≥ 1)	0.25 (S)	0.5 (MS)	0.25 (S)
Ciprofloxacin (S ≤ 0.06; MS = 0.12–0.5; R ≥ 1)	16 (R)	8 (R)	16 (R)

^*^Methodology Guidelines MUK 4.2.1890–04. Determining Microorganism Susceptibility to Antibacterial Medications: Recommended Practices. Moscow: Federal Center of State Sanitary and Epidemiological Surveillance, Ministry of Health of the Russian Federation, 2004. P. 91.

^**^As the Methodology Guidelines MUK 4.2.1890–04 contains no criteria for azithromycin susceptibility, the evaluation was carried out using the criteria of the European Committee for Antimicrobial Susceptibility Testing (www.eucast.org)

An analysis of the susceptibility of the studied strains to six antimicrobials previously or currently recommended for the treatment of gonococcal infection showed either resistance or moderate susceptibility of the strains to benzylpenicillin, tetracycline, and ciprofloxacin. *N. gonorrhoeae *41/15/003 was additionally characterized by moderate susceptibility to azithromycin (*Table 1*).

**Sequencing of the *N. gonorrhoeae* genome**

The genomic DNA was isolated from *N. gonorrhoeae *1-colony cultures on chocolate agar supplemented with 1% ISOVitalex additive (Becton Dickinson, USA) incubated during 18–24 h using a PROBA-NK kit (DN-Technology, Russia). A library of random DNA fragments 400–700 bp long was prepared using the standard GS Rapid Library protocol. The libraries were amplified in emulsion PCR using GS Junior emPCR kit. Whole-genome sequencing was carried out by the 454 pyrosequencing technology on a GS Junior system (Roche, Switzerland). Each genome was sequenced using CS Junior Titanium Sequencing Kit in an individual sequencing run. The mean coverage of genomes was ≥ 20.

**Bioinformatic analysis methods**

FastQC and Trimmomatic was used for the quality control and trimming. Remaining reads were assembled using Spades. Assembled *N. gonorrhoeae *genomes were annotated using Prokka [12], employing the Swiss- Prot (high-priority species-level references) and UniProt-KB (genus-level references) datasets of amino acid sequences. Prior to annotation, the UniProt-KB dataset was clustered using CD-HIT [13], with 90% identity of protein length and amino acid sequences. Ribosomal RNA genes were annotated using Barrnap (https://github.com/tseemann/barrnap). Aragorn was used to annotate tRNAs. IslandViewer 4 [14] was used to detect genomic islands.

The search for the genetic determinants of antibiotic resistance in *N. gonorrhoeae *was performed by RGI analysis in the Comprehensive Antibiotic Resistance Database (CARD) [15].

The NGMASTER software was employed for NG-MAST sequence typing of *N. gonorrhoeae *[16]; multilocus MLST sequence typing was performed using the SRST2 software [17]. Orthologous protein groups belonging to the genus *Neisseria *were inferred by OrthoFinder [18] from annotated protein sequences of the analyzed isolates and 24 previously sequenced *N. gonorrhoeae *strains, as well as NCBI reference annotations of *N. meningitidis*, *N. lactamica *and *N. elongata*. Each of the 790 selected orthologous groups was subjected to multiple sequence alignment using MAFFT [19] with 2,000 refinement iterations. Each alignment was masked using Gblocks. Masked and concatenated alignments were used to infer a maximum likelihood phylogenetic tree of *N. gonorrhoeae *in RAxML [20] with 500 bootstrap replicates. The FigTree graphical viewer was used to visualize the tree with projected support values (http:// tree.bio.ed.ac.uk/software/figtree/).

In order to characterize the overall evolution of the coding sequences and the sequences associated with the formation of antimicrobial resistance, we formed two sets of concatenated multiple sequence alignments of the corresponding orthogroups and calculated the *p-*distance within each set. Matching between the cells of two resulting matrices was estimated using the Spearman’s rank correlation coefficient.

## RESULTS AND DISCUSSION

**Overall characterization of the *N. gonorrhoeae* genomes**

The assembly of the genome sequences of the studied *N. gonorrhoeae *strains revealed a circular chromosome 2,223,815 to 2,271,213 bp long with G+C content of 52.5–52.7% in each genome (*Table 2*).

**Table 2:** Overall characteristics of the genomes of the analyzed N. gonorrhoeae strains

Characteristics	Strains		
	20/15/004	41/15/003	19/15/005
Genome size, bp	2271213	2236575	2223815
G+C content, %	52.6	52.5	52.7
Number of protein-coding genes	2448	2297	2293
Number of protein-coding genes with known function	1332	1266	1281
Number of the 16S-23S-5S rRNA genes	4	4	4
Number of tRNA genes	49	49	47
Number of tmRNA genes	1	1	1
Number of “genomic islands” along the bacterial chromosome	12	22	17
Plasmid size, bp	4556	5233	5266
Plasmid coverage depth to chromosome coverage depth	18.4	12.3	33.6

The total number of identified open reading frames was 2,448 (*N. gonorrhoeae *20/15/004), 2,297 (*N. gonorrhoeae *41/15/003), and 2,293 (*N. gonorrhoeae *19/15/005). Among the genes, 1,332 (54.4%), 1,266 (55.1%), and 1,281 (55.9%) open reading frames were annotated as protein-coding genes with the known function, respectively.

Each of the analyzed genomes was found to contain 49 (*N. gonorrhoeae *20/15/004 and *N. gonorrhoeae *41/15/003) or 47 (*N. gonorrhoeae *19/15/005) tRNA genes, one tmRNA gene, and four copies of 16S-23S-5S rRNA operon.

Cryptic plasmids varying in length from 4,556 (*N. gonorrhoeae *20/15/004) to 5,266 bp (*N. gonorrhoeae *19/15/005), carrying the relaxosome gene *MobC *(typical of conjugative plasmids), genes of the cryptic plasmid proteins A, B, and C, and five open reading frames with unknown functions were identified for each strain. The rate of mean plasmid coverage to the mean chromosome coverage in 20/15/004 and 41/15/003 strains yielded 18.4 and 12.3, respectively, versus 33.6 in 19/15/005 strain, thus characterizing the revealed plasmids to be present in multiple copies (> 10 copies per cell).

In general, the first stage of bioinformatic analysis revealed the analyzed genomes to be similar to those of the reference FA1090 (GenBank: AE004969) and some other previously sequenced *N. gonorrhoeae *strains [21], including those of the ST 1407 genogroup [22]. A degree of quantitative variations between the compared genomes could be caused by the high genome plasticity conventionally attributed to the presence of prophages, transposons, and the insertion sequence elements IS110 and IS1016 [23], which were revealed within genomic islands in all three *N. gonorrhoeae *strains in a substantially high amount (*Table 2*).

The sequences of the *de novo *assembled genomes were deposited into the GenBank NCBI database with IDs NTCT00000000 (*N. gonorrhoeae *20/15/004), NTCS00000000 (*N. gonorrhoeae *41/15/003), and NTCU00000000 (*N. gonorrhoeae *19/15/005).

**Genetic determinants of antimicrobial resistance in *N. gonorrhoeae***

The next step in the bioinformatic analysis involved finding and studying four groups of genes encoding 1) the enzymes responsible for the inactivation of antibiotics or the modification of their targets, 2) the protein targets with mutations reducing their affinity for the respective antimicrobials, 3) the transport proteins delivering antibiotics into bacterial cell, and 4) the antibiotic efflux systems (*Table 3*).

**Table 3:** Genetic determinants of antimicrobial resistance in N. gonorrhoeae

Genes (proteins)	Resistance to antimicrobials	Genes and nucleotide polymorphisms (amino acid substitutions)		
		20/15/004	41/15/003	19/15/005
Enzymes responsible for inactivation of antibiotics or modification of their targets				
blaTEM (β-lactamase)	β-lactams	-	-	-
ermA/B/C/F (rRNA methylases)	macrolides	-	-	-
Proteins targeting antibiotics				
ponA (PBP_1_)	β-lactams	L421P	L421P	L421P
penA (PBP_2_)	β-lactams	I312M V316T F504L N512Y G545S	I312M V316T F504L N512Y G545S	F504L P551S
tetM	tetracyclines	-	-	-
rpsJ (S10)	tetracyclines	V57M	V57M	V57M
rpsE (S5)	spectinomycin	wt	wt	wt
rrs (16S RNA)	spectinomycin	wt	wt	wt
rrl (23S RNA)	macrolides	wt	wt	wt
rplD (L4)	macrolides	wt	wt	wt
rplV (L22)	macrolides	wt	wt	wt
gyrA	fluoroquinolones	S91F D95G	S91F D95G	S91F D95G
parC	fluoroquinolones	S87R E91A	S87R E91A	wt
parE	fluoroquinolones	wt	wt	wt
Transport proteins delivering antibiotics into the cell				
penB /porB (PorB1b)	β-lactams tetracyclines	G120K A121N	G120K A121N	G120D
penC/pilQ	β-lactams	wt	wt	wt
Enzymatic antibiotic efflux systems				
mtrCDE	β-lactams tetracyclines macrolides	wt	wt	wt
mtrRpro		A35del	A35del	wt
macAB	macrolides	wt	wt	wt
macABpro		wt	wt	wt
mefA	macrolides	wt	wt	wt
norM	fluoroquinolones	wt	wt	wt
norMpro		wt	wt	wt

Note: “-” – the gene was not found; “wt” – wild-type sequence.

**Resistance determinants for β-lactams (penicillins and cephalosporins)**

In the analyzed *N. gonorrhoeae *sequences, the results didn’t reveal both *blaTEM-1 *or *blaTEM-135 *genes encoding the β-lactamase enzyme, which hydrolyzes the lactam ring in the penicillin molecule and other antimicrobials with a similar structure [24].

On the other hand, the sequences of chromosomal genes encoding penicillin binding proteins (PBPs) were found to carry a number of nucleotide substitutions that significantly reduce susceptibility to β-lactams. Thus, all three strains carried the mutation L421P in *ponA *gene encoding PBP1, resulting in reduced affinity to penicillins compared to the wild-type protein [25]. Even greater changes were detected in the *N. gonorrhoeae *20/15/004 and *N. gonorrhoeae *41/15/003 *penA *gene sequences (encoding PBP2), reflecting the concept of mosaic-like structure formed due to a genetic recombination with synanthropic commensals, such as *N. cinerea *and *N. perflava* [26]. More amino acid substitutions, namely F504L, N512Y, and G545S, were found in the C-terminal region of PBP2, and they significantly reduced the binding rate between the peptidyl transferase center and the antimicrobial molecule imparing functionally important conformational changes [27]. Moreover, two other amino acid substitutions, I312M and V316T, are considered to be associated with the formation of cephalosporin resistance, especially in combination with the G545S substitution [28]. On behalf of this, two amino acid substitutions, F504L and P551S, which reduce the level of acylation of PBP2, as much as other known mutations in the C-terminal region of this protein characterizing the *penA *allele in *N. gonorrhoeae *19/15/005 [29].

The *penB *gene encoding the outer membrane porin PorB1b and currently denoted as *porB*, is also involved in the emergence of resistance to β-lactams in *N. gonorrhoeae*. The amino acid substitutions G120K and A121N in this protein reducing membrane permeability for hydrophilic antibiotics [30] were detected in *N. gonorrhoeae *20/15/004 and *N. gonorrhoeae *41/15/003, whereas the *N. gonorrhoeae *19/15/005 strain carried a single G120D substitution.

The *pilQ *gene (previously known as *penC*) is another studied gene attributed to the resistance to β-lactams and other hydrophilic antibiotics in *N. gonorrhoeae*. The additional pores formed in the outer membrane by the product of this gene enable antibiotic diffusion into the periplasmic space of the bacterial cell. Mutation in the triplet 666(Gly) or complete deletion of *pilQ *gene can increase the level of antibiotic resistance in *N. gonorrhoeae*, especially when accompanied with the resistance determinants in the *penA *and *penB *genes [31]. However, this gene was wild-type in the analyzed genomes.

To finalize the analysis of the resistance determinants to β-lactams in *N. gonorrhoeae*, we must point out the three tangled genes present in all the analyzed strains. These genes lie within the multiple transferable resistance (Mtr) locus controlled by the MtrR repressor and encode the MtrC-MtrD-MtrE efflux pump system. The analysis of the *mtrR *promoter region revealed deletion A35del, which ensured elimination of this repression, together with an increase in antibiotic resistance [32]. This mutation was found in the *N. gonorrhoeae *strain 20/15/004 (ST 1407) and in the structurally related *N. gonorrhoeae *strain 41/15/003 (ST 12556) with a similar structure of *porB *gene. This is in accordance with the hypothesis that gonococci of the 1407 NG-MAST genogroup possess the above discussed mechanism of antibiotic resistance [5].

**Tetracycline resistance determinants**

A point mutation causing the amino acid substitution V57M in ribosomal protein S10 of the 30S ribosomal subunit was found in the chromosomal *rpsJ *gene in all three analyzed genomes. This substitution disrupts binding of the antimicrobial to ribosome and allows one to concider this as the mechanism underlying the resistance to tetracyclines in *N. gonorrhoeae *[33].

On the other hand, the genomes of the three analyzed strains were found to carry no Dutch or American variants of *tetM *gene [34]; the protein products of the variants interfere with the elongation factors EF-G and EF-Tu and make the ribosome inaccessible for interaction with the antimicrobial.

In turn, the nonspecific mechanisms of resistance to tetracyclines (identically to β-lactams) in the analyzed strains involved mutations in PorB1b protein and MtrC-MtrD-MtrE efflux system, which is an effective supplement to the specific resistance mechanism caused by a mutation in *rpsJ *gene [33].

**Spectinomycin resistance determinants**

The nucleotide sequence in *rrs *gene matched the sequence of a wild-type gene with cytosine at position 1186 (corresponding to position 1192 in *Escherichia coli*); this is the key nucleotide in the binding site for the interaction of aminocyclitols with helix 34 of 16S RNA [35].

Another analyzed chromosomal determinant was *rpsE *gene, which encodes the ribosomal protein S5 of 30S ribosomal subunit; mutations in this protein can cause spectinomycin resistance, although *rrs *gene remains to be wild-type. However, searching for the possible amino acid substitutions T24P [36] and K28E, as well as for deletion of codon 27 (Val) [37], confirmed the wild-type allele of *rpsE *gene.

**Macrolide resistance determinants**

Neither of the analyzed genomes contained *ermA/B/C/F *gene cluster, which codes rRNA methylases modifying the macrolide binding sites of the 23S rRNA molecule.

The results of the search for A2059G and C2611T mutations in *rrl *gene, which disrupt the interaction between macrolide antibiotics and their target (the peptidyl transferase center) in domain V of 23rRNA [38], indicate that all three strains were wild-type.

The products of the *rplD *and *rplV *genes (ribosomal proteins L4 and L22) that bind to domain I of 23S rRNA and likewise have multiple binding sites for the other domains of 23S rRNA, were also wild-type. Mutations in the L4 and L22 proteins alter the conformation of domains II, III, and V; this may affect the susceptibility of microorganisms to macrolides [39].

The analysis of *N. gonorrhoeae *resistance to macrolides revealed that all the three genomes possess functional alleles of the *macA *and *macB *genes encoding the MacA–MacB complex, which specifically recognizes and removes the antimicrobial from the periplasm of bacterial cells [40]. However, an analysis of position -10 of promoters of these genes characterized them as wild-type without efflux pump overexpression. The *mefA *gene encoding another transport protein responsible for macrolide resistance [41] was also wild-type in the studied genomes.

**Fluoroquinolone resistance determinants**

The search for chromosomal mutations defining fluoroquinolone resistance in *N. gonorrhoeae *included the analysis of quinolone resistance-determining regions (QRDRs) in the *gyrA*, *parC, *and *parE *genes encoding DNA gyrase subunit A, as well as the topoisomerase IV subunits C and E, the fluoroquinolone targets.

In the all three strains, *gyrA *gene was found to contain single-nucleotide polymorphisms TCC → TTC and GAC → GGC causing the S91F and D95G amino acid substitutions associated with fluoroquinolone resistance in *N. gonorrhoeae *[42].

The *parC *gene in *N. gonorrhoeae *20/15/004 and *N. gonorrhoeae *41/15/003 possessed wild-type triplets (86(D) and 88(S)), indicating a double mutation with S87R and E91A amino acid substitutions. This mutation, together with the changes in DNA gyrase, significantly modified the structure of the so-called quinolone-binding pocket [43], making impossible the interaction between the antimicrobial and the target. The analysis of all four amino acid substitutions demonstrated the presence of the wild-type *parC *gene in the genome of *N. gonorrhoeae *19/15/005. Wild-type *parE *gene was present in all the analyzed *N. gonorrhoeae *trains.

Characterization of the nonspecific mechanism of fluoroquinolone resistance in *N. gonorrhoeae *revealed that each of the studied genomes contained one functional copy of the *norM *gene encoding the membrane transporter that removes cationic antimicrobials from the bacterial cell [44]. Also, the promoter region at position -35 was wild-type, suggesting no additional efflux pump overexpression.

**Molecular typing and phylogenetic analysis of *N. gonorrhoeae***

The variation of antibiotic resistance determinants in the studied genomes of *N. gonorrhoeae *strains required a revision of their phylogenetic relationship. In this regard, we used a combination of NG-MAST and multilocus sequence typing (MLST) results to plot a dendrogram comparing the entire set of housekeeping genes.

The findings of the sequenced genomes analysis using the NGMASTER software [16] revealed a complete coincidence with the initial data referring the analyzed strains to the genogroups ST 1407, ST 12556, and ST 12450 (*Table 4*).

Furthermore, the SRST2 analysis [17] of the nucleotide sequences of conserved *abcZ*, *adk, aroE, fumC, gdh*, *pdhC*, and *pgm *genes [45] revealed *N. gonorrhoeae *strains 20/15/004 and 41/15/003 to refer to the MLST type 1901, which relates to the epidemic high-risk ST 1407 according to the NG-MAST sequence typing data [5]. On the other hand, four of seven alleles of N. gonorrhoeae 19/15/005 characterized this strain as the MLST type 6721. This has never been mentioned in publications concerning antimicrobial resistance, and the phylogenetic relation with ST 1407 has never been described.

**Table 4:** Results of molecular mapping of N. gonorrhoeae

Genes and the encoded molecular types (NG-MAST and MLST)	Sequence types and numbers of alleles of N. gonorrhoeae strains		
	20/15/004	41/15/003	19/15/005
NG-MAST	1407	12556	12450
porB	908	971	931
tbpB	110	6	2097
MLST	1901	1901	6721
abcZ	109	109	126
adk	39	39	39
aroE	170	170	67
fumC	111	111	111
gdh	148	148	146
pdhC	153	153	153
pgm	65	65	133

The phylogenetic relationship between the strains were evaluated by comparing the entire set of 790 orthologous groups of housekeeping genes in *Neisseriaceae *family members. We used additional data from the NCBI database on the genomes of the *N. meningitidis *and 24 *N. gonorrhoeae *strains, including the reference strain FA 19, five previously sequenced ST 1407 strains, and 18 strains of other NG-MAST types. *Figure* shows the phylogenetic tree designed by maximum likelihood estimation using the RAxML program [20] (the Gamma substitution model in combination with the BLOSUM62 scoring matrix). In order to assess the relation between the evolution of neutral coding sequences and the sequences involved in the formation of antibiotic resistance, we compared this phylogenetic tree to the dendrogram plotted for the orthologous groups associated with antimicrobial resistance.

The resulting data show an extremely high genotype likelihood between the strains 20/15/004 (ST 1407) and 41/15/003 (ST 12556), according to the set of housekeeping genes combined to a single cluster with the other previously sequenced representatives of the genogroups ST 1407, ST 6146, and ST 3520. Hence, the results of the study confirmed the hypothesis of a close phylogenetic relation between the analyzed strains. The first strain emerged in the population of *N. gonorrhoeae *in Russia after putative international migration, while the other one has diverged from their common ancestor as a result of single mutation events. On the other hand, the third analyzed strain, 19/15/005 (ST 12450), was substantially distant from the ST 1407 genogroup on the phylogenetic tree of housekeeping genes and exhibited no close genotypic relation to any of the formed genogroups. Therefore, there was a prominent discordance between the primary molecular typing data and the results of whole-genome sequencing of this strain, indicating that antibiotic-resistant strains in the current Russian *N. gonorrhoeae *population are polyphyletic.

Comparison of the concatenated multiple alignments of neutral coding sequences and sequences associated with the formation of antimicrobial resistance revealed a statistically significant (*p * < 0.001) positive correlation (the Pearson ’s correlation coefficient = 0.44). In particular, this correlation was demonstrated for representatives of genogroup ST 1407 (including 20/15/004 and 41/15/003 strains), except for ST 3520. The latter sequence type was also an isolate characterized by a mosaic structure of *penA *gene [46] but with probably its own independently developed mechanisms of antimicrobial resistance. The analysis of *N. gonorrhoeae *19/15/005 (ST 12450) has confirmed the relatively independent phylogenetic position of this strain.

## CONCLUSIONS

Whole-genome sequencing of three *N. gonorrhoeae *strains isolated in Russia in 2015 was performed [10]. According to the NG-MAST results, these strains possess multiple antimicrobial resistance and preliminarily were referred to the epidemic high-risk genogroup ST 1407, spread currently worldwide [6, 8].

The analysis of the genomes revealed that they essentially pertain to the reference *N. gonorrhoeae *strain FA1090 [21] and previously sequenced representatives of the ST 1407 genogroup [22]. The revealed absence of *ermA/B/C/F *genes and presence of wild-type alleles of the *rpsE*, *rrs*, *rrl*, *rplD*, *rplV*, *macAB, *and *mefA *genes explain the susceptibility of the studied strains to certain groups of antimicrobials, as mutations in these genes are associated with the emergence of resistance to aminocyclitols (spectinomycin) and macrolides (azithromycin). However, the conjugative resistance determinants (*blaTEM, tetM*) were absent in the genomes and the *penC/pilQ*, *parE, *and *norM *alleles were shown to be wild-type, whereas single or multiple nucleotide substitutions were identified in the genes encoding targets for β-lactams (*ponA, penA*), tetracyclines (*rpsJ*), and fluoroquinolones (*gyrA, parC*). The additional mutations were found in *porB *gene and the promoter of *mtrR *gene and, therefore, they nonspecifically reduced the susceptibility to antimicrobials due to membrane permeability decreasing and efflux pump overexpression. Hence, the findings in the results of the whole-genome sequencing coincide with the preliminary results of the phenotypic analysis. The analysis of genetic resistance determinants allowed us to predict susceptibility or resistance to certain antimicrobial groups, but no MIC values could be inferred from these data. In particular, although the genotypes of the *N. gonorrhoeae *strains 20/15/004 (ST 1407) and 41/15/003 (ST12556) are similar, the increased resistance of the latter from 2 up to 4-fold to penicillin, ceftriaxone, tetracycline, and azithromycin remains unexplained. Otherwise, the different mutation spectra of the genes involved in emergence of antimicrobial resistance in *N. gonorrhoeae *20/15/004 (ST 1407) and 19/15/005 (ST 12450) resulted in similar parameters of antimicrobial resistance. This indicates the importance of the search for additional mechanisms of antimicrobial resistance in *N. gonorrhoeae *and developing computer algorithms to ensure sufficient consistency between the results of genotypic and phenotypic analyses [47].

**Figure:**
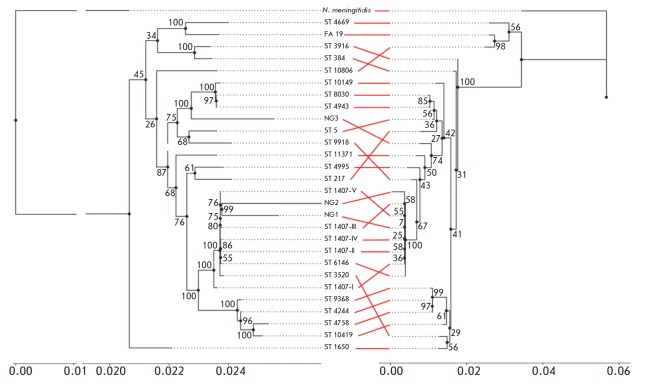
Phylogenic mapping of the analyzed isolates (NG1 = 20/15/004; NG2 = 41/15/003; NG3 = 19/15/005) referring to the other N. gonorrhoeae strains studied previously. Phylogenic mapping was performed by comparing the housekeeping genes (left-hand side) and orthologous groups related to the emergence of antimicrobial resistance (right-hand side). The correspondence of genome numbering in the NCBI database: ST 1407-I = SRR3349203; ST 1407-II = SRR3349826; ST 1407-III = SRR3357181; ST 1407-IV = SRR3357194; ST 1407-V = PMC3486552; ST 5 = SRR3349550; ST 217 = SRR3349568; ST 384 = SRR3350138; ST 1650 = SRR3343502; ST 3520 = SRR3357021; ST 3916 = SRR3343568; ST 4244 = SRR3350168; ST 4669 = SRR1661263; ST 4758 = SRR3343553; ST 4943 = SRR3349831; ST 4995 = SRR3349564; ST 6146 = SRR3349969; ST 8030 = SRR3349209; ST 9368 = SRR2736298; ST 9918 = SRR3349572; ST 10149 = SRR3349522; ST 10419 = SRR3343607; ST 10806 = SRR3349206; ST 11371 = SRR3349525. The length of the branches of the phylogenetic tree (X axis) corresponds to the number of expected amino acid substitutions per position. The values at branch nodes represent the level of branch support. Red lines connect the homonymous branches

Another important result of this study was the revision of the phylogenetic association of the analyzed clinical isolates, compared to initially formulated findings acording to the NG-MAST data. This study has confirmed a high homology between the *N. gonorrhoeae *20/15/004 (ST 1407) and *N. gonorrhoeae *41/15/003 (ST 12556) genomes; the latter probably diverged in Russia from a representative of the internationally spread ST 1407 clone. On the other hand, *N. gonorrhoeae *strain 19/15/005 (ST 12450) was an example of phenotypic and genotypic convergence with independent formation of antimicrobial resistance by its own, although partially similar, mechanisms. This result indicates the antibiotic resistant strains in the population of *N. gonorrhoeae *in Russia as being polyphyletic, and this give reasons for further research in order to identify and consider an expanded list of epidemic high-risk genotypes.

## Abbreviations

MIC: minimal inhibitory concentration
MLST: multilocus sequence typing
NG-MAST: Neisseria gonorrhoeae multi-antigen sequence typing
PBP: penicillin-binding protein
QRDR: quinolone resistance-determining regions
ST: sequence type
